# The association between surgeon grade and radiographic implant alignment following oxford unicompartmental knee replacement

**DOI:** 10.1007/s00402-025-05973-y

**Published:** 2025-07-03

**Authors:** Muhamed M. Farhan-Alanie, James Miller, Alastair Stephens, Tsun Yu Kwan, Tarek Boutefnouchet

**Affiliations:** 1 Warwick Medical School, University of Warwick, Coventry, UK; 2 Department of Trauma & Orthopaedics, University Hospital Coventry & Warwickshire NHS Trust, University Hospital Coventry, Coventry, UK; 3 Department of Trauma & Orthopaedics, Heartlands Hospital, University Hospitals Birmingham NHS Trust, Birmingham, UK

**Keywords:** Knee arthroplasty, Implant alignment, Revision, Surgical training

## Abstract

**Introduction:**

Unicompartmental knee replacement (UKR) is a technically challenging operation. Component alignment can influence implant longevity and knee function post-operatively. This study aimed to investigate implant alignment following UKR performed by consultants compared to trainees.

**Methods:**

100 Oxford UKRs performed by trainees and consultants were analysed. Two blinded surgeons independently assessed post-operative knee radiographs on four parameters: flexion/extension of femoral component, posterior slope of tibial component, and varus/valgus of femoral and tibial components. Logistic regression was performed to predict the probability of implant malpositioning outside the optimal position range.

**Results:**

Median varus/valgus angles for femoral components did not differ significantly between trainees and consultants (p = 0.92), nor did the angles for tibial components (p = 0.43). Posterior tibial slope measurements showed a significant difference between trainees and consultants (7.08° [IQR 5.2–9.30], and 5.35° [IQR 2.65–7.05], respectively; p < 0.01). Median flexion/extension angles for femoral components also differed significantly between trainees and consultants (−14.45° [IQR −19.2 to −9.85] and −10.2°[IQR −13.55 to −6.95], respectively; p < 0.01). A greater proportion of implants positioned by trainees were classified as outliers for this parameter (46% versus 20%, p < 0.01; aOR 5.39, 95% CI 2.05–14.18, p < 0.01). However, no differences in the proportion of outliers was found when trainees were directly supervised by consultants (p = 0.73).

**Conclusions:**

Trainees achieved adequate component alignment within optimal ranges for most parameters however were more prone to positioning the femoral component in excessive flexion. Greater emphasis on achieving optimal flexion/extension positioning of the femoral component during surgical training and direct supervision may improve the outcomes of patients undergoing an Oxford UKR by trainees.

## Introduction

The number of unicompartmental knee replacement (UKR) procedures performed annually has steadily increased and is projected to continue rising [1]. This trend is partly due to increased recognition that UKR is suitable in approximately half of patients with knee osteoarthritis, which is the most common indication for this operation [2, 3]. Outcomes following UKR are influenced by patient selection, surgical technique, and component positioning [4, 5]. UKR surgery is widely considered by surgeons as a procedure that is relatively more technically challenging to perform compared to other arthroplasty surgeries [6, 7]. This is partly because the exposure is limited and precise positioning of the components is more difficult to achieve [7, 8]. Within the current training system in England, trainee surgeons develop their surgical skills under the supervision of consultant surgeons. When trainee surgeons are deemed appropriately competent by their consultant, they are provided the opportunity to perform the operation. Due to the steep learning curve of UKR, post-operative outcomes, including function and implant longevity, may vary depending on whether the procedure is performed by trainees or consultants [9]. Only one previous study has been completed which has compared survivorship of UKR performed by trainees and consultants [10, 11]. The study analysed 1,084 procedures and found no significant differences in revision or exchange of any part of the prosthetic components between the patient groups. However, this study is underpowered to detect differences in revision given this ism a rare event. Furthermore, the need for revision may not manifest until many years following the index procedure, and studies investigating this outcome require a long period of follow up. In contrast, component alignment can be considered a surrogate predictor of implant longevity and knee function post-operatively, and has been validated for both these outcomes [4, 12–18]. As such, this study aimed to compare UKR implant alignment between procedures performed by consultants and trainees. This study also investigated differences in all-cause reoperation and intraoperative complications.

## Methods

### Patient identification and eligibility criteria

We searched the departmental arthroplasty database at [BLINDED] for patients aged ≥ 18 years who underwent a primary unicompartmental knee replacement. The first 50 consecutive procedures performed by consultant surgeons and surgical trainees from inception of the database to the search date were included for analysis (1st January 2012–21st April 2023, respectively).

Inclusion criteria were medial tibiofemoral joint UKR performed exclusively for osteoarthritis, which is the most common indication for this procedure [19]. Operations involving additional procedures such as ligament reconstruction or performed with the assistance of computer assisted technologies were not eligible for inclusion to specifically assess the impact of the surgeon’s grade on UKR implant alignment. We restricted procedures to those performed using the uncemented version of a single prosthesis brand (Oxford twin-peg partial knee; Zimmer Biomet). This implant was selected due to its prevalent and ongoing use by surgeons in our institution.

### Exposures and outcomes of interest

The exposures of interest were whether the UKR procedure was performed by a consultant surgeon or surgical trainees including fellow. Primary outcomes were coronal and sagittal implant alignment of the femoral and tibial components, specifically varus/valgus angle of femoral and tibial component, flexion/extension angle of femoral component, and posterior slope angle of the tibial component [4, 12–18]. A sub-analysis, stratifying by whether the trainee surgeon was directly or indirectly supervised by a consultant surgeon, was performed. Direct supervision refers to the presence of a scrubbed consultant in the operating theatre, whereas indirect supervision denotes the consultant is outside the operating theatre but nearby, ready and available to offer support if needed. Secondary outcomes were re-operation for all-causes and their indications, and intra-operative complications.

### Data collection including radiographic assessment

Data was collected on several variables including patient age at time of index procedure, sex, American Society of Anaesthesiologists (ASA) grade, body mass index (BMI), year of surgery, side of procedure, grade of surgeon who performed the procedure, presence of a scrubbed consultant during UKR performed by trainee, intra-operative complications, re-operation, indication for re-operation, and mortality. Data on mortality is linked to the National Health Service of England ‘Spine’ healthcare network which aggregates information from various data sources.

**Fig. 1:**
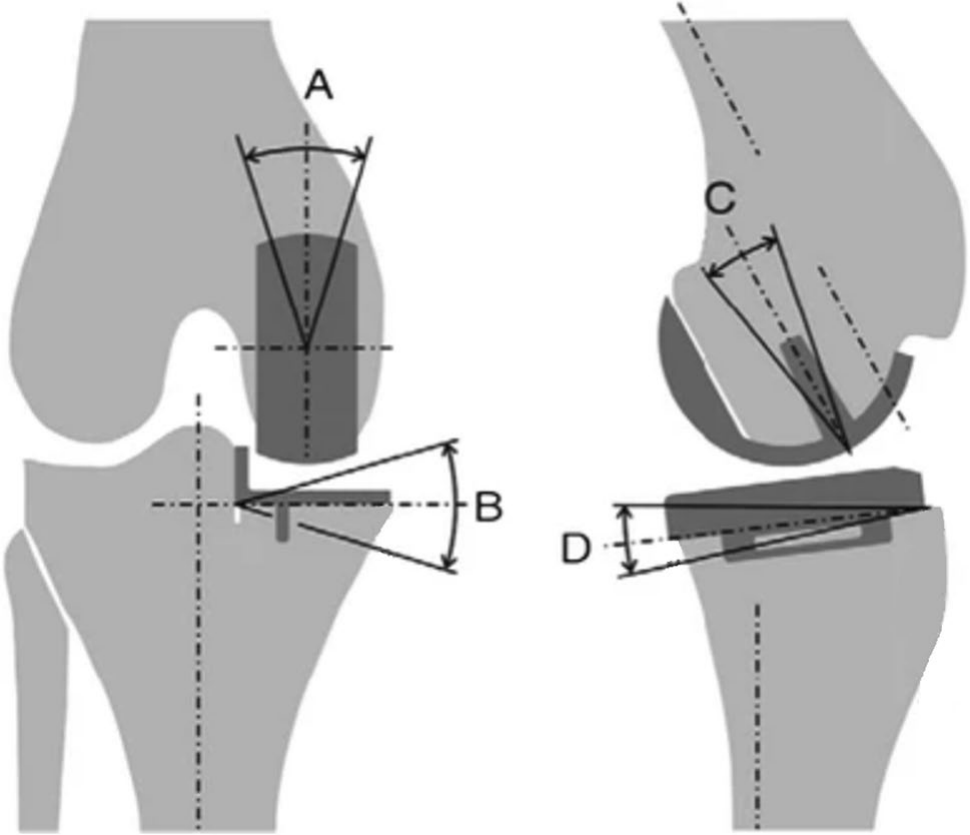
Measurements obtained from radiological analysis. **A** Femoral component valgus/varus. **B** Tibial component valgus/varus. **C** Femoral component flexion/extension. **D** Tibial component posterior slope. Figure reproduced from Pandit et al. [45], ‘Aetiology of lateral progression of arthritis following Oxford medial unicompartmental knee replacement: a case–control study’, published in Musculoskeletal Surgery, under the Creative Commons Attribution License (CC BY)

For the radiographic assessment, the earliest available post-operative knee radiographs for patients were located and patient identifiers were removed to conceal the identity and grade of the surgeon who performed the operation. Anteroposterior and lateral radiographs of the knee were then converted from DICOM to JPEG image format, and sent to two authors (surgeons) for blinded, independent assessment (AS and JM). Radiograph images were assessed on four parameters: varus/valgus angle of femoral and tibial component, flexion/extension angle of femoral component, and posterior slope angle of tibial component. These parameters, with optimal ranges detailed in Table 1, were measured by both authors according to documented instructions [4, 20–22]. The varus/valgus alignment of the tibial and femoral components were assessed using the long axis of the tibia, while flexion/extension of the femoral component and slope of the tibial component were assessed relative to the posterior femoral and tibial cortices, respectively (Fig. 1). This radiographic assessment technique was based on methods used in previous research [23, 24]. Implant alignment angles were measured using Weasis Dicom Medical Viewer software (v4.1.2, 2009–2023) [25]. Radiographs deemed to be unsuitable for evaluation by either reviewer due to their quality were excluded. In such instances, both reviewers were provided with additional radiographs for assessment from an alternative UKR procedure performed.

**Table 1:** Oxford UKR described ranges for the radiographic parameters used to assess implant alignment

Radiographic parameter	Optimum position range
Femoral component: varus/valgus angle (Anteroposterior radiograph)	< 10.0° varus to < 10.0° valgus (relative to mechanical axis)
Femoral component: flexion/extension angle (Lateral radiograph)	15.0° flexion to < 0° extension
Tibial component: varus/valgus angle (Anteroposterior radiograph)	< 5.0° varus to < 5.0° valgus (perpendicular to tibial axis)
Tibial component: posterior slope angle (Lateral radiograph)	> 2° to < 12° posterior tilt (relative to tibial axis)

### Statistical analysis

The study’s sample size was based on a previous study which reported a standard deviation of 2.51° for the measurement of varus/valgus angle of the tibial component among experienced surgeons using the same implants [26]. This parameter was selected to calculate the required sample size given it had the narrowest optimum position range. Considering a minimally clinically important difference of 5⁰ degrees for this parameter, and setting power at 0.90 and significance level at 0.05, a total sample size of 14 patients was needed.

Categorical variables were summarised as frequencies and percentages. Parametric data was reported using mean and standard deviation (SD). Non-parametric data was reported as median and interquartile range (IQR). Analyses were conducted using suitable parametric and non-parametric tests. The average value of the measurement data of the four radiographic implant alignment parameters that were determined by the two assessors were used in the analyses. The intraclass correlation coefficient (ICC) for each radiographic parameter was computed and interpreted using the guideline by Koo et al. [27]. Multivariable logistic regression was performed to predict the probability of implant malpositioning outside the optimal position range, adjusting for year of surgery. All-cause reoperation was analysed using Kaplan–Meier estimates to account for censoring due to death or absence of experiencing a re-operation event. A Cox proportional hazards model was used to assess for differences in all-cause reoperation. Proportional hazards assumption was checked through visual assessment of plots and statistical testing of scaled Schoenfeld residuals. 95% confidence intervals (CI) are presented, and statistical significance was set at p < 0.05. Analyses were performed using Stata software (version 18.0, StataCorp LLC, College Station, Texas, USA, 1985–2023).

## Results

### Patient characteristics

**Fig. 2:**
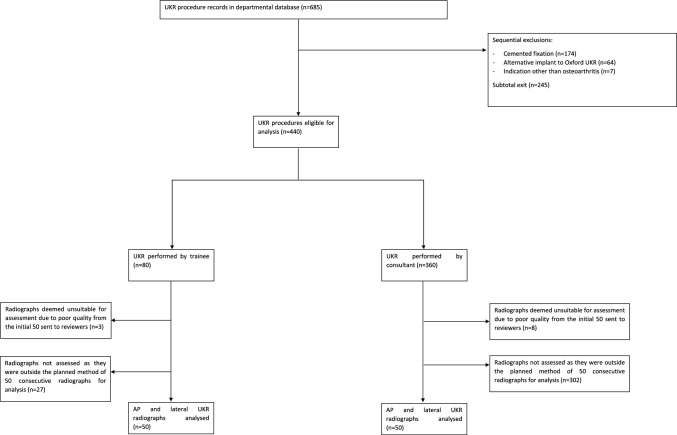
Flow diagram illustrating the process of inclusion and exclusion of procedure records

Figure 2 illustrates the study flow diagram.

The characteristics of the two patient groups were similar however there was a difference in the time periods of UKR procedures analysed between the two groups. A relatively larger proportion of procedures were performed by trainees during a later time period (Table 2).

**Table 2:** Characteristics of patients undergoing primary UKR

	Consultant	Trainee	p-value
Mean age, years (SD)	62.89 (8.78)	63.92 (11.02)	0.61
Median year of procedure (IQR)	2013 (2012—2014)	2013 (2012—2016)	0.027
Sex (%)			
Female	21 (42)	2 (44)	0.84
Male	29 (58)	28 (56)	
Side (%)			
Left	22 (44)	28 (56)	0.23
Right	28 (56)	22 (44)	
ASA classification (%)			
1	19 (38)	13 (26)	0.08
2	31 (62)	33 (66)	
3	0	4 (8)	
4	0	0	
5	0	0	
BMI			
Mean (SD)	29.49 (3.60)	30.52 (4.94)	0.25
Availability (%)	47 (94%)	48 (96%)	0.65

### Radiographic implant alignment analysis

The ICC ranged from 0.77 to 0.96 indicating good or excellent agreement for all implant alignment parameters measurements [27]. The specific ICC for each parameter is reported in Table 3.

**Table 3:** ICCs of radiographic parameters

Radiographic parameter	ICC	95% CI
Flexion/extension angle femoral component	0.96	0.95–0.98
Varus/valgus angle femoral component	0.87	0.80–0.91
Flexion/extension tibial component	0.77	0.66–0.84
Varus/valgus angle tibial component	0.81	0.71–0.87

**Fig. 3:**
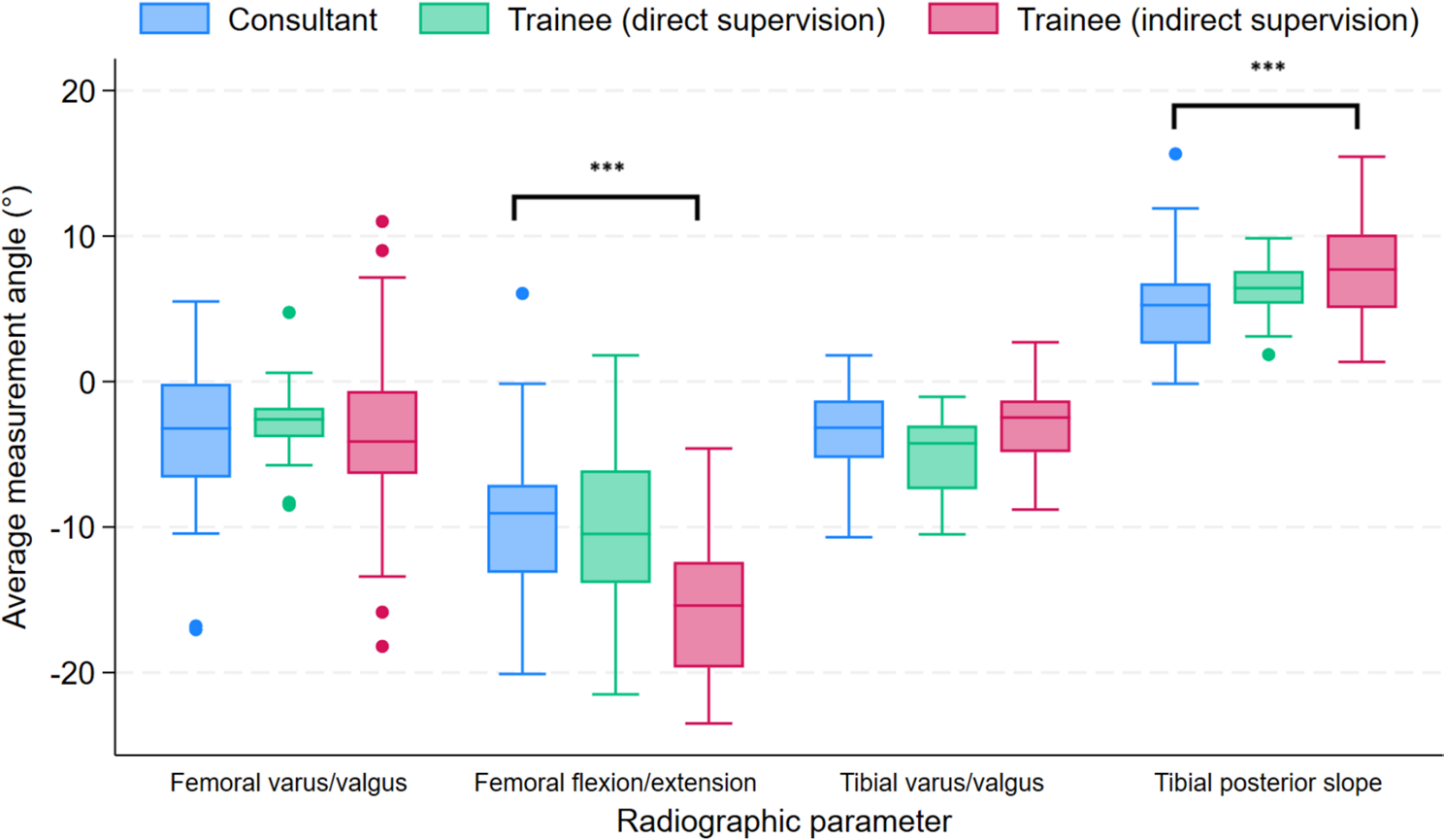
Box plot displaying the distribution of measured angles for various radiographic parameters assessed in UKRs performed by consultants and trainees under direct and indirect supervision

**Fig. 4:**
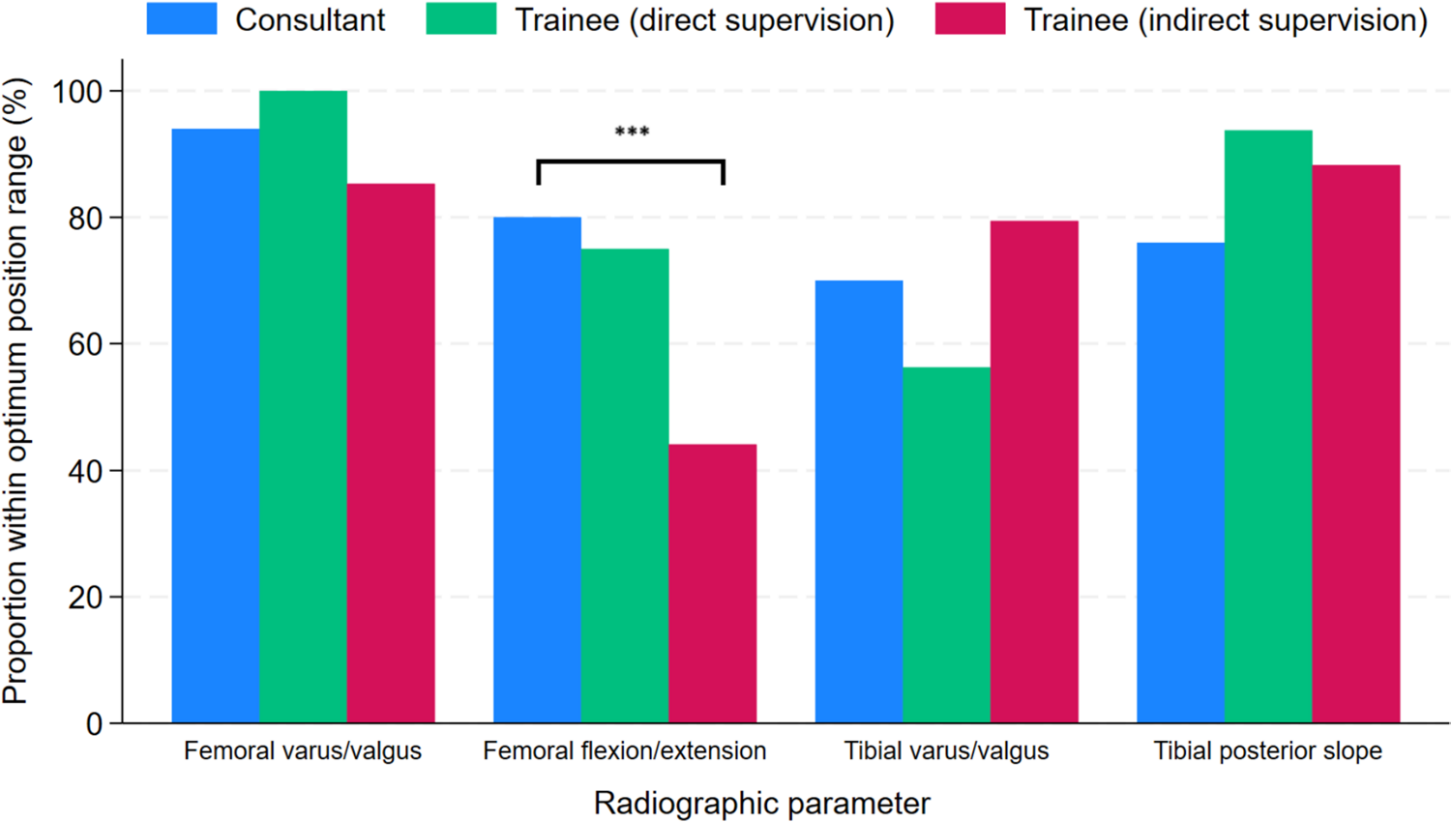
Proportion of implants within optimum position range for various radiographic parameters assessed in UKRs performed by consultants and trainees under direct and indirect supervision

Table 4 details the median values of the angles and proportion of outliers for the four radiographic implant parameters of UKRs performed by consultants and trainees. It also includes the results when stratifying trainee surgeons by type of consultant supervision. This analysis showed no significant differences in the proportion of outlier components between UKRs performed by consultants and trainees under direct supervision for all four radiographic parameters. However, a greater proportion of outliers was found for the flexion/extension angle of the femoral component in the subgroup of trainees who received indirect consultant supervision. Figures 3 and 4 illustrate the distribution of measured angles and proportion of implants within the optimum position range for the various radiographic parameters assessed in the UKRs performed by consultants and trainees under direct and indirect supervision, respectively.

**Table 4:** Comparison of radiographic implant alignment between UKR procedures performed by consultants and trainees including stratification by type of consultant supervision

Radiographic parameter	Outcome measure	Consultant (n = 50)	Trainee (n = 50)	p-value^a^	Trainee (direct consultant supervision) (n = 16)	p-value^b^	Trainee (indirect consultant supervision) (n = 34)	p-value^c^
Flexion (−)/extension (+) angle femoral component	Median angle (IQR)	−9.05° (−13.15 to −7.1)	−14.45° (−19.2 to −9.85)	< 0.01	−10.48° (−13.85 to −6.1)	0.74	−15.4° (−19.65 to −12.4)	< 0.01
	Number of satisfactory ranges (%)	40 (80)	27 (54)	0.01	12 (75)	0.73	15 (44.12)	< 0.01
	Number of outliers (%)	10 (20)	23 (46)		4 (25)		19 (55.88)	
	Adjusted odds ratio (95% CI)	1.0 [reference]	5.39 (2.05 to 14.18)	< 0.01	3.36 (0.58 to 19.33)	0.18	5.96 (2.17 to 16.35)	< 0.01
Varus (−)/valgus (+) angle femoral component	Median angle (IQR)	−3.23° (−6.6 to −0.15)	−3.30° (−5.75 to −1.3)	0.83	−2.6° (−3.83 to −1.8)	0.76	−4.13° (−6.35 to −6.50)	0.63
	Number of satisfactory ranges (%)	47 (94)	45 (9)	0.46	16 (100)	1.00	29 (85.29)	0.26
	Number of outliers (%)	3 (6)	5 (10)		0		5 (14.71)	
	Adjusted odds ratio (95% CI)	1.0 [reference]	1.86 (0.40 to 8.68)	0.43	~	−	2.55 (0.55 to 11.74)	0.23
Posterior (+) slope tibial component	Median angle (IQR)	5.25° (2.6 to 6.75)	7.08° (5.2 to 9.30)	< 0.01	6.43° (5.35 to 7.60)	0.09	7.7° (5.05 to 10.1)	< 0.01
	Number of satisfactory ranges (%)	38 (76)	45 (90)	0.06	15 (93.75)	0.16	30 (88.24)	0.26
	Number of outliers (%)	12 (24)	5 (10)		1 (6.25)		4 (11.76)	
	Adjusted odds ratio (95% CI)	1.0 [reference]	0.35 (0.11 to 1.17)	0.09	0.20 (0.01 to 2.85)	0.23	0.37 (0.10 to 1.34)	0.13
Varus (−)/valgus (+) angle tibial component	Median angle (IQR)	−3.18° (−5.25 to −1.3)	−3.30° (−5.8 to −1.7)	0.94	−4.25° (−7.4 to −3.03)	0.06	−2.48° (−4.85 to −1.3)	0.30
	Number of satisfactory ranges (%)	35 (70)	36 (72)	0.83	9 (56.25)	0.37	27 (79.41)	0.45
	Number of outliers (%)	15 (30)	14 (28)		7 (43.75)		7 (20.59)	
	Adjusted odds ratio (95% CI)	1.0 [reference]	0.90 (0.36 to 2.25)	0.83	4.09 (0.78 to 21.36)	0.10	0.64 (0.22 to 1.80)	0.40

^a^Comparison between consultants and all trainees

^b^Comparison between consultants and trainees (direct consultant supervision)

^c^Comparison between consultants and residents (indirect consultant supervision) ~ Unable to compute due to nil outliers in trainee subgroup

### Intra-operative complications

There was one intra-operative complication among the 100 UKR procedures. This occurred during a surgery being performed by a consultant grade surgeon and was reported as a tibial cut error in the sagittal plane that drifted to the lateral aspect of the knee. This was addressed by adjusting the implant position and using bone graft.

### Re-operation for all-causes

**Fig. 5:**
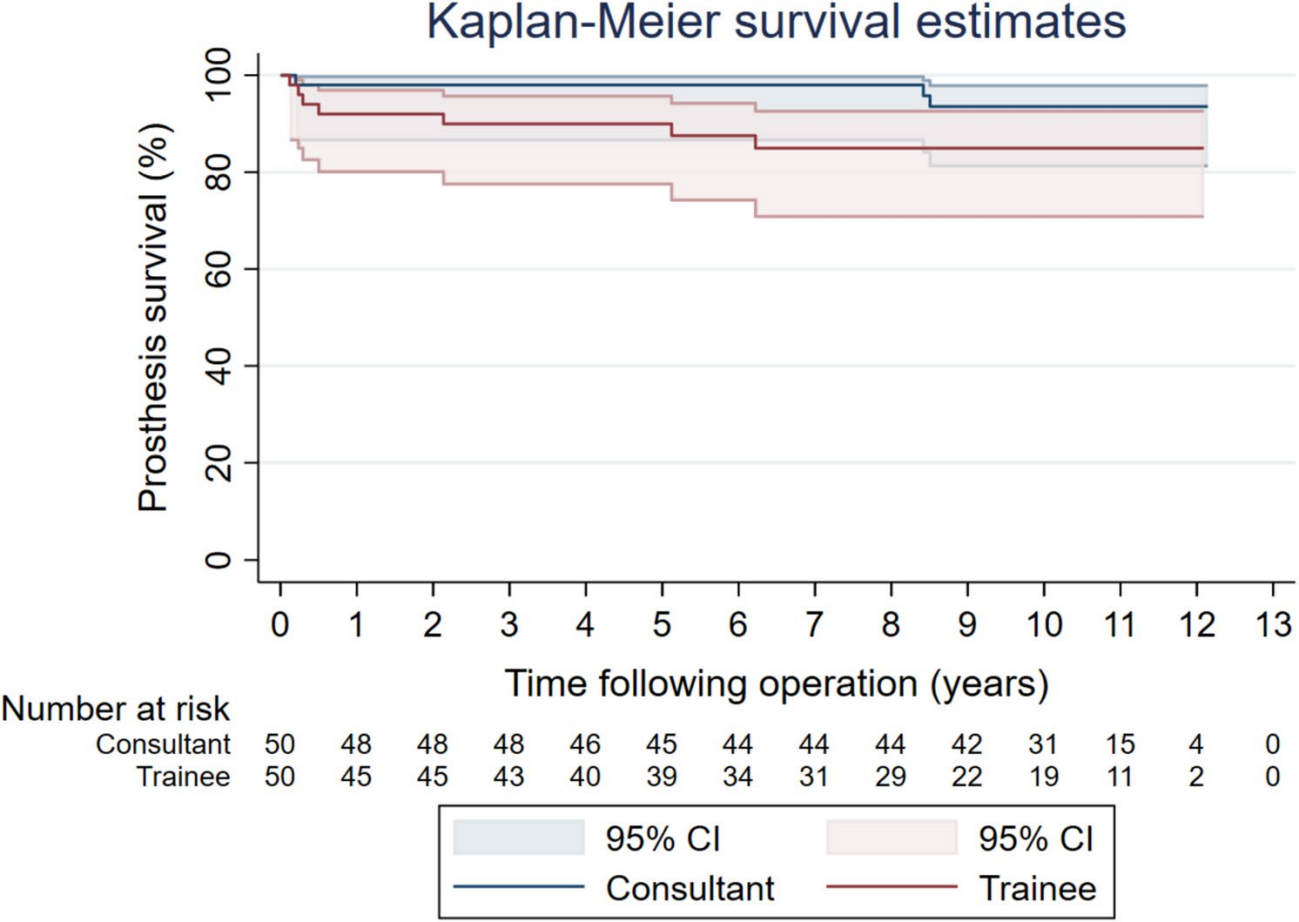
Re-operation for all-causes following primary UKR performed by consultant versus trainee

There were three and seven reoperations following UKR performed by trainees and consultants respectively. There were no differences in risk of re-operation for all-causes between groups (hazard ratio 2.80, 95% CI 0.72–10.92, p = 0.14) (Fig. 5). Table 5 details the causes for re-operation.

**Table 5:** Causes for re-operation following UKR performed by consultants and trainees. Figures in parentheses denote timing in days from index operation

	Consultant	Trainee	p-value
Progression of osteoarthritis requiring revision to TKR^a^	2 (3075 and 3107)	1 (2272)	0.251
DAIR for PJI^b^	0	2 (44 and 1871)	
Revision to TKR due to periprosthetic fracture	1 (73)	0	
Dislocated meniscal bearing^c^	0	2 (183 and 780)	
Manipulation under anaesthesia for stiffness	0	1 (86)	
Revision to TKR due to pain	0	1 (780)	
Total	3	7	

^b^Prosthetic joint infection

^c^One procedure required revision to TKR and other procedure involved exchange to thicker bearing

## Discussion

The present study identified that trainees achieved adequate component alignment within optimal ranges for most investigated parameters however were more prone to malposition the femoral components of cementless medial Oxford UKR in excessive flexion in the sagittal plane compared to consultant surgeons. Analysis investigating the effect of consultant supervision type found this difference to be present only when trainees were indirectly supervised. Furthermore, we found that regardless of the type of consultant supervision, there were no differences in the proportion of implant positions that were outliers for the other parameters investigated including posterior slope of the tibial component and varus/valgus angles of the femoral and tibial components. Although significant differences were found between the median values for posterior slope of the tibial component between trainees and consultants, both these values fell within the optimum range. We also found there were no differences in all-cause risk of re-operation between patient groups however this analysis was underpowered. These findings have important implications for surgical training in UKR surgery and potentially suggests that greater emphasis on achieving precise sagittal alignment of the femoral component during training may help address this observed challenge. The observed differences in sagittal alignment of the femoral component, alongside the absence of differences in the other parameters assessed between consultant surgeons and indirectly supervised trainees, may reflect varying learning curves across different parts of the procedure. Furthermore, the limited exposure from a minimally invasive incision may particularly impair visualisation and increase the difficulty of achieving precise femoral component placement in the sagittal plane. Additionally, precise alignment of the tibial component may be less challenging, as the lower leg is not typically draped, providing a clearer visual reference during the procedure. This study is the first to investigate UKR implant alignment parameters variation between trainee and consultant surgeons. Our findings for component positioning are similar to previous studies that have investigated these same radiographic parameters using the Oxford unicompartmental knee implant. The exact values of the radiographic parameters of the UKRs performed by the consultant surgeons in these studies were very similar and the proportion of implants that were considered outliers had ranged from 0 to 52% [28–34]. Alvand et al. conducted a single-centre randomised controlled trial comparing patient specific instrumentation to conventionally instrumented Oxford UKR, and reported on post-operative radiological results of procedures performed by four consultant surgeons. Measured values of the radiographs of procedures performed using conventional instrumentation were within two degrees of our study’s results for all four parameters investigated [34]. Our study’s ICC values for the radiographic analysis are also similar to those previously conducted [35]. For revision outcomes, two previous studies have compared results between trainees and consultants. Bottomley et al. carried out a single-centre comparative cohort study involving 1084 UKR procedures [10]. Trainees were supervised by a scrubbed consultant surgeon for 48% of their procedures, and no differences were found in implant survival between consultants and trainees (p = 0.30). However, there were differences in indications for revision between groups with all revisions for bearing dislocation occurring in procedures performed by the trainee group. Our study also found that re-operations performed due to bearing dislocation were limited to patients who had a UKR performed by a trainee however this was not statistically significant. Another single-centre study by Alvand et al. analysed 273 UKR procedures and using the reported data, we estimated crude revision rates at five years post-operatively to be approximately 1% and 2.92% for procedures performed by trainees and consultants respectively [36]. This data represents a relative risk of 0.34 (95% CI 0.04–3.02, p = 0.34).

The strengths of this study include the use of two independent and blinded assessors which helped minimise detection bias for the analysis of radiographs. Suboptimal x-rays were excluded ensuring only those suitable for analysis were included. The high ICC values obtained for all four radiographic parameters confirmed there was good agreement between the two assessors. Selection bias was minimised by analysing x-rays of consecutive patients since the inception of our arthroplasty database. Also, the characteristics of patients included in our study were very similar between UKR procedures performed by trainees and consultants allowing comparable groups. In addition, the analyses of radiographic implant alignment, including when stratifying by the type of consultant supervision for procedures performed by trainees, were both appropriately powered. The evaluation of the effect of the type of consultant supervision on this outcome enhanced the study by allowing an improved understanding of the association between this factor and trainee performance. Furthermore, we focused exclusively on a single, commonly used prosthesis brand and patients who underwent medial tibiofemoral osteoarthritis to avoid confounding due to these factors. We also performed logistic regression analyses adjusting for year of surgery to account for confounding related to changes over the study time period, such as the introduction of microplasty instrumentation.

There are limitations to our study including those inherent to any radiological analysis. We also investigated select radiographic parameters, omitting others such as the fit and rotation of the tibial component, and patients post-operative leg alignment. These parameters are better assessed using computed tomography and long leg radiographs, which were unavailable. However, it has been shown that there is good correlation between the standard knee and long leg radiographs [37]. We restricted procedures to those performed using a single prosthesis brand however different prostheses have their own set of instruments with potentially different learning curves. This means it is possible our study results may not be generalisable to all UKR procedures. We categorised trainees at different stages of their training into a single group. However, there may be performance differences within this group attributable to varying levels of experience with UKR surgery. Conversely, the group of trainees who performed the UKR procedure without direct consultant oversight likely had similar levels of experience given their consultant deemed them competent to carry out the procedure in this manner. However we cannot exclude the possibility of confounding by indication where relatively straightforward cases may have been deliberately assigned to trainees to perform. It is worth highlighting however that indications for UKR procedures are relatively strict, which may have helped ensure similarity in unmeasured characteristics between patient groups. Although our study focused on procedures performed at a single hospital, most trainee surgeons had previously undergone training in UKR arthroplasty at other hospitals, which improves the generalisability of our study’s findings. It is also important to mention that the training model and experience of trainee surgeons performing UKR at other hospitals may differ, limiting the generalisability of our study’s results. We also restricted the study population to medial UKR and results may not be generalisable to lateral UKR which may be considered technically more challenging. For outcome re-operations, we are unable to exclude the possibility that patients may have experienced a re-operation at a different institution. Furthermore, this analysis was underpowered to detect differences in this outcome between groups.

It is established that malposition of the femoral component is a common error even amongst experienced surgeons [33]. However, it is unknown whether excessive femoral component flexion that occurred in the UKRs performed by trainees has clinically important effects on functional outcomes as this was not investigated in our study. Additionally, previous research has investigated the effects of implant positioning on extreme intervals within the optimum range only [23]. However, excessive femoral component flexion is theoretically believed to increase the risk of meniscal bearing dislocation [38–40]. This could possibly be a contributing factor in helping to explain why re-operations for this complication occurred only in procedures performed by trainees in our study, as well as the significant differences in revision for this indication in a previous study [10, 41]. Lastly, it is worth highlighting that trainees successfully avoided potentially more clinically significant errors, such as valgus alignment of the tibial component, which is believed to promote development of lateral compartment osteoarthritis and is a common cause of revision [14, 42]. In contrast, excessive varus of the tibial component has been shown to potentially increase creep and wear of the polyethylene bearing [12]. Similarly, trainees also managed to avoid excessive posterior tibial slope, which has been shown to impair cruciate ligament function and compromise knee stability, and is associated with increased pain [43, 44].

In conclusion, this study has identified a possible signal suggesting that indirectly supervised trainees may malposition the femoral component of UKR in excessive flexion. However, trainees were proficient in achieving optimal component alignment for the other parameters evaluated. Given the paucity of research on this topic, the potential educational benefits for trainees, and the implications for improving patient outcomes, further investigation of this topic is warranted. Future studies should involve larger sample sizes using a multicentre approach and where possible analyse computed tomography images to investigate outcomes including re-operation for all-causes, patient-reported outcome measures evaluating physical function, and precision of component positioning.
